# Social Isolation as a Precipitating Factor for Charles Bonnet Syndrome in a Patient with Mild Visual Deterioration

**DOI:** 10.3390/reports7030065

**Published:** 2024-08-02

**Authors:** Shriya Prakash Bhat, Abeezar Shipchandler, Chris Tokunaga

**Affiliations:** 1 Department of Infectious Disease and Microbiome, Broad Institute, Cambridge, MA 02142, USA; 2 Medical Director, Hospital Medicine, Baylor Scott and White, Plano, TX 75093, USA; ship@bswhealth.org; 3 Psychiatry, Baylor Scott and White, Temple, TX 76508, USA; chris.tokunaga@bswhealth.org

**Keywords:** Charles Bonnet Syndrome (CBS), visual hallucinations, social isolation

## Abstract

Charles Bonnet Syndrome (CBS) is characterized by complex visual hallucinations in individuals with acute vision loss, typically affecting older adults. Most CBS cases are observed in patients with sudden severe visual impairment; however, there are limited reports of CBS occurring in individuals with mild visual deterioration, particularly when confounded by sensory deprivation and social isolation. Here, we report a case of CBS in a female in her early 80s, who experienced vivid visual hallucinations during a period of prolonged social isolation following a hip surgery. Although symptomatology initially presented as delirium and psychosis, the diagnosis of CBS was confirmed after a neuropsychiatric evaluation and ophthalmologic exam, which showed mild deterioration in the patient’s macular degeneration, combined with a social history of reduced independence and increased isolation. The case highlights the lesser-known role of sudden lifestyle changes and reduced cognitive engagement in CBS development in cases of mild visual impairment. It also suggests that cognitive and social stimulation may play a crucial role in CBS management, which has limited reports in the current literature.

## 1. Introduction

Charles Bonnet Syndrome (CBS) is characterized by vivid visual hallucinations in individuals experiencing vision loss—notably, in the absence of psychosis, delirium, drug overdose, stroke, or other identifiable neurologic causes [1]. Typically, CBS manifests in older adults between the ages of 70 and 85, coinciding with increased prevalence with common underlying conditions causing vision loss, including age-related macular degeneration, glaucoma, and cerebral infarction [2]. Hallucinations related to CBS are not simple illusions; rather, they consist of complex and detailed images of people, animals, or intricate patterns [2]. Those experiencing CBS have insight into the fact that these hallucinations are not real—this differentiates it from other conditions that involve visual hallucinations. It is also important to note that CBS does not occur in individuals with congenital blindness.

Most previously reported cases of CBS have been observed in individuals with severe acute vision loss (Table 1). However, there is a lack of consensus on the severity of loss necessary for appropriate diagnosis [3,4,5]. In less common cases of mild visual impairment, as in our patient, numerous compounding factors are thought to increase the risk of CBS, including cognitive impairment, cerebrovascular disease, cortical atrophy on brain imaging, and social deprivation [6,7,8,9]. In particular, social isolation and inactivity have been found to be associated with CBS development, although its precise role in CBS genesis remains unclear. Recent studies have found exacerbations in CBS symptomatology during periods of isolation and loneliness during the COVID-19 pandemic, potentially due to increased cognitive load that could heighten the vividness and frequency of hallucinations [10]. Conversely, during periods of hospitalization, some CBS patients experienced no hallucinations, likely due to increased social engagement [11]. Studies have also implicated living alone with the onset of symptoms, as well as the number and quality of social interactions and feelings of belonging, suggesting that a lack of sensory input and mental stimulation resulting from isolation can aggravate hallucinations [12,13]. We present a less common case in an elderly female with mild visual impairment who experienced vivid hallucinations, likely attributed to similar experiences of social isolation and inactivity. We hope to raise awareness and provide clearer diagnostic criteria for CBS in these less common cases to enhance understanding and promote early and accurate diagnosis.

## 2. Case Presentation

A Caucasian female in her early 80s with a history of macular degeneration, senile cataracts, congenital strabismus, Duane’s syndrome, and various cardiovascular and endocrine conditions presented to the emergency department with reported visual hallucinations. Our patient, a college-educated retired elementary school teacher, had been widowed for 14 years and maintained an independent lifestyle until experiencing a fall two months prior to the current incident. After the fall, the patient had become homebound and dependent on her son for transportation and housekeeping, necessitating home health care for physical therapy. The patient’s family reported a sudden altered mental status due to visual hallucinations. These included encounters of peculiar children with missing limbs who seemed “not quite alive”, visions of children’s’ heads during meals, and a bizarre incident of a doll-like figure with a little girl inside the restroom. The patient recounted being informed of a movie involving young children which she never saw, as well as mistaking a toy for a real gorilla during physical therapy, speculating that it might have been intended to uplift her spirits. Notably, these hallucinations would resolve upon closing her eyes for a few minutes.

The onset of symptoms occurred two weeks prior to presentation, with no reported psychosocial stressors or history of dementia, neurological illness, or mental health problems. The patient denied substance use. She was on Levothyroxine, Valsartan, Carvedilol, Hydralazine, Myrbetriq, and baby Aspirin. Physical examination and comprehensive neurological assessment were unremarkable, and cognitive examination was within normal limits for the patient’s age, with no neurologic deficits. A previous emergency department visit a week prior for similar symptoms ruled out stroke and other serious causes, diagnosing a mild urinary tract infection that was treated with Cefuroxime, but proved ineffective. Laboratory investigations, including hematological, biochemical, and microbiological tests, yielded normal results. A chest X-ray, CT of the head, brain MRI, and brain and neck MRA were normal given the patient’s age and no unusual abnormalities were found. The patient was initially prescribed Quetiapine 25 mg for sleep disturbance and possible psychosis. However, due to the lack of neurologic, infectious, or metabolic causes, our patient was referred to geriatric psychiatry for evaluation of possible early dementia or geriatric psychosis.

The patient underwent a Mental Status Evaluation, which demonstrated normal speech, mood, affect, thought processes, and thought content, with no indications of delusions or paranoia. The patient also denied any suicidal or homicidal ideation during her evaluation. Her cognition was grossly intact, with fair insight, impulse control, judgment, memory, attention, and concentration, and she was oriented to person, place, and time. The patient’s Quetiapine was discontinued, and she was referred to ophthalmology as an outpatient. Ophthalmologic examination included visual acuity testing and a fundoscopic examination. The patient presented with stable ptosis and strabismus (well-adjusted to prism in glasses), a stable intraocular lens, and Duane’s syndrome in the left eye. Visual acuity for distant and near vision was 20/60 (OD) and 20/25 (OS) and 20/50 (OD) and 20/70 (OS), respectively. Funduscopic examination of the right eye revealed slight progression of the circumscribed area of geographic atrophy, indicative of mild advancement in macular degeneration. The patient received a new prescription for corrective glasses, and visual acuity for distant vision and near vision was corrected to 20/25 (OD) and 0/20 (OS) and 20/30 (OD) and 20/30 (OS), respectively (Table 2). The diagnosis of Charles Bonnet Syndrome was subsequently made based on clinical findings and the patient’s social history.

**Figure 1:**
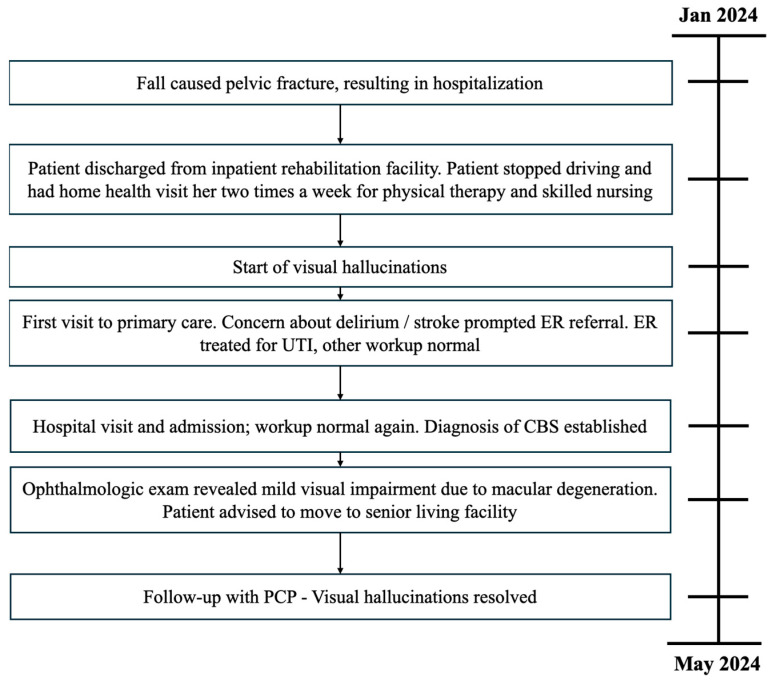
Timeline of patient case.

Ophthalmology recommended relocation to a senior living facility to enhance social interaction. During a six-week follow-up with her primary care physician, the patient, now residing in a senior living facility, reported her well-being improved and had no recurrence of visual hallucinations since then (Figure 1).

## 3. Discussion

Charles Bonnet Syndrome (CBS) is a commonly overlooked but prevalent cause of complex visual hallucinations affecting 10% to 15% of individuals with visual impairment [10]. These hallucinations, often referred to as release hallucinations, are thought to arise from the brain’s response to reduced visual input [11]. Key symptoms include vivid, well-formed, and often elaborate visual hallucinations in patients with underlying acquired visual impairment but in the absence of psychosis, impaired sensorium, dementia, intoxication, metabolic derangement, or focal neurological illness.

CBS diagnosis was not initially considered following the patient’s first emergency department presentation due to the absence of severe visual impairment, which is typically associated with reported cases of CBS. However, the patient’s recent lifestyle changes—transitioning from near-complete independence and social engagement to being socially isolated and homebound following her hip surgery—combined with visual deterioration and unremarkable neurological assessment, prompted consideration of CBS. We hypothesize that the significant reduction in social engagement and cognitive stimulation during the patient’s prolonged recovery period, coupled with mild visual impairment, may have increased the likelihood of endogenous visual stimulus generation by the brain, manifesting as complex visual hallucinations. These hallucinations, including visions of non-living children and inanimate objects appearing animate, were consistent with those typically reported in CBS cases. This case suggests that factors beyond the severity of visual impairment, such as sudden lifestyle changes and reduced cognitive engagement, may contribute to CBS development [8,12,13]. Common differential diagnoses are presented in Table 3. In cases of complex hallucinations with mild visual impairment, clinically, we emphasize eliciting a history of social isolation or reduced environmental stimuli as a precipitating factor to support the diagnosis. We further emphasize the importance of physicians considering environmental factors in both the etiology and treatment of CBS: in such cases, clinicians should consider cognitive and social stimulation in CBS management such as recommending senior living facilities and community activities and support groups.

One limitation of this report is the lack of longitudinal data; continued follow-up with the patient will ensure the sustained impact of social engagement on CBS symptoms over time. Additionally, it should be noted that the assessment of inactivity and loneliness is subjective and cannot be quantified with standardized measures and should be assessed on a case-by-case basis. Although a comprehensive evaluation, including a brain MRI, toxicologic studies, and laboratory investigation, is necessary to rule out secondary causes, heightened awareness of CBS, especially in cases of mild visual impairment, would help facilitate earlier detection and management by clinicians, thus mitigating patient destress, preventing multiple ER visits and/or hospitalizations, and improving patient outcomes.

## 4. Conclusions

Our patient represents a classic case of Charles Bonnet Syndrome, but with mild visual deterioration compared to severe acute vision loss, which is attributed to most cases. Although the patient’s visual impairment was not significant, her prescription was adjusted slightly to better meet her needs. We hypothesize that inactivity and loneliness contributed to the onset of symptoms—when moved to a senior living facility, the patient regained social activity and, since then, has not experienced any further episodes of hallucinations. This case highlights the potential role of social isolation in CBS development and suggests that social engagement may be an important component of CBS management in individuals with mild visual deterioration. We underscore the importance of considering environmental factors in CBS etiology and treatment.

## Figures and Tables

**Table 1:** Summary of typical CBS cases reported in the literature.

Authors, Year	Age	Vision Loss Etiology	Visual Acuity	Key Findings	Treatment
Jackson et al., 2009 [1]	69	Exudative age-related macular degeneration	20/70 (OD), 20/50 (OS)	Abrupt change in vision 16 months prior to the onset of visual hallucinations	Intraocular ranibizumab injections to prevent visual deterioration
Jan et al., 2012 [2]	86	Open-angle glaucoma, macular degeneration, cataracts	20/50 (OD) 20/100 (OS)	Abrupt decline in vision in a week, 30/30 Mini Mental Status Exam (MMSE)	Visual correction
Maruzairi et al., 2022 [3]	63	Bilateral retinal detachment, cataract s/p intraocular lens implant in right eye	None provided	Visual hallucinations associated with persecutory delusion due to worsening vision in right eye, symptoms resolved upon closing eyes and prayer	Treatments of Quetiapine and Zolpidem
Kompella et al., 2022 [4]	67	Diabetic retinopathy and bilateral sensorineural hearing loss	None provided	Veteran experiencing visual and musical hallucinations for few days, associated depression and mild dementi	Reassurance; trial of antipsychotics worsened symptoms
Kelson et al., 2022 [5]	93	Bilateral cataracts	20/50 (OD) 20/60 (OS)	Visual hallucinations with mild cognitive impairment, sudden 2 week decline in visual acuity, 25/30 MMSE	Motivational interviewing, supportive psychotherapy, assisted living facility
Voit et al., 2021 [7]	68	Right-sided ptosis, blepharoplasty, infarct in the right posterior occipital lobe	20/40 (OD) 20/60 (OS)	Hallucinations in left visual field, subacute infarct in the right posterior occipital lobe	Neurology and ophthalmology follow-up at tertiary center
Jacob et al., 2004 [14]	87	Registered blind due to advanced macular degeneration	1/60 (OD) 1/60 (OS)	Confused for early dementia	Reassurance
Lang et al., 2006 [15]	78	Registered blind	Hand motion	Four-week history of depression, hallucinations occurring for one year	Treatment of Venlafaxine

**Table 2:** Visual acuity and fundoscopic examination findings for right and left eye.

	Right Eye (OD)	Left Eye (OS)
Visual Acuity Testing		
Distant	20/60	20/25
Near	20/50	20/70
Fundoscopic Exam		
Cup to Disc Ratio (C)	0.3 (Normal)	0.3 (Normal)
Optic Nerve	Peri-papillary atrophy present	Peri-papillary atrophy present
Vitreous	(−) Shafer’s sign (negative)	(−) Shafer’s sign (negative)
Retinal Exam	2+ Drusen2+ Retinal Pigment Epithelium (RPE) Changes 1+ Geographic Atrophy Vessels within normal limits Retina is flat and attached 360°	1+ Drusen 1+ Retinal Pigment Epithelium (RPE) Changes Vessels within normal limits Retina is flat and attached 360°

**Table 3:** Differential diagnoses for patients presenting with visual hallucinations.

Etiology	Typical Features	Duration of Hallucinations	Triggers for Hallucinations	Insight	Associated Symptoms
Charles Bonnet Syndrome (CBS) [11,16]	Complex and detailed images of people, animals, or intricate patterns	Variable—usually minutes	Sensory deprivation	Intact	Visual impairment with either cerebrovascular disease, cortical atrophy on brain imaging, or social deprivation
Migraine [16]	Simple	Minutes to hours	Lack of sleep, menses, certain foods, stress	Intact	Headache, nausea, vomiting, photophobia
Seizures [17]	Simple	Seconds	None	Intact	Convulsions, post-ictal headache
Retinal Pathology [18]	Simple, such as flashing lights	Seconds	Vitreous detachment triggered usually by valsalva	Intact	Possible vision loss and abnormal fundoscopic exam
Parkinson’ Disease [19]	Simple or complex	Mutes	None	Variable, related to cognitive status	Parkinsonism
Psychiatric Illness [20]	Complex	Variable	None	Absent	Disordered thoughts, delusions
Alcohol Withdrawal [21]	Complex	Persistent until treated	Stopping alcohol suddenly	Impaired	Confusion, agitation
Narcolepsy [22]	Complex	Minutes	Falling to or awakening from sleep	Intact	Sleep disorders such as excessive daytime sleepiness, cataplexy, sleep paralysis

## Data Availability

The original data presented in the study are included in the article, further inquiries can be directed to the corresponding author.

## References

[1] Jackson M.L., Ferencz J. (2009). Cases: Charles Bonnet syndrome: Visual loss and hallucinations. CMAJ.

[2] Jan T., Del Castillo J. (2012). Visual hallucinations: Charles bonnet syndrome. West. J. Emerg. Med..

[3] Maruzairi H., Joo C.L. (2022). A Case Report on Charles Bonnet Syndrome. Iran. J. Psychiatry.

[4] Kompella S., Kaushal S., Khan S.A., Alvarez Villalba C.L. (2022). A Case Report and Review: Charles Bonnet Syndrome Plus With Dementia. HCA Healthc. J. Med..

[5] Kelson M., Santos T., Athanasios A., Fitzsimmons A. (2022). Out of sight, am I losing my mind? A case report on Visual ReleHallucinations—Charles Bonnet Syndrome. Psychiatry Res. Case Rep..

[6] Russell G., Burns A. (2014). Charles Bonnet syndrome and cognitive impairment: A systematic review. Int. Psychogeriatr..

[7] Voit M., Jerusik B., Chu J. (2021). Charles Bonnet Syndrome as Another Cause of Visual Hallucinations. Cureus.

[8] Jones L., Ditzel-Finn L., Enoch J., Moosajee M. (2021). An overview of psychological and social factors in Charles Bonnet syndrome. Ther. Adv. Ophthalmol..

[9] Reichert D.P., Seriès P., Storkey A.J. (2013). Charles Bonnet syndrome: Evidence for a generative model in the cortex?. PLoS Comput. Biol..

[10] Jones L., Ditzel-Finn L., Potts J., Moosajee M. (2021). Exacerbation of visual hallucinations in Charles Bonnet syndrome due to the social implications of COVID-19. BMJ Open Ophthalmol..

[11] Menon G.J., Rahman I., Menon S.J., Dutton G.N. (2003). Complex visual hallucinations in the visually impaired: The Charles Bonnet Syndrome. Surv. Ophthalmol..

[12] Holroyd S., Rabins P.V., Finkelstein D., Lavrisha M. (1994). Visual hallucinations in patients from an ophthalmology clinic and medical clinic population. J. Nerv. Ment. Dis..

[13] Nicholson N.R. (2009). Social isolation in older adults: An evolutionary concept analysis. J. Adv. Nurs..

[14] Jacob A., Prasad S., Boggild M., Chandratre S. (2004). Charles Bonnet syndrome-elderly people and visual hallucinations. BMJ.

[15] Lang U.E., Stogowski D., Schulze D., Domula M., Schmidt E., GaLLinat J., Tugtekin S.M., Felber W. (2006). Charles Bonnet Syndrome: Successful treatment of visual hallucinations due to vision loss with selective serotonin reuptake inhibitors. J. Psychopharmacol..

[16] Teunisse R.J., Zitman F.G., Raes D.C. (1994). Clinical evaluation of 14 patients with the Charles Bonnet syndrome (isolated visual hallucinations). Compr. Psychiatry.

[17] Panayiotopoulos C.P. (1999). Visual phenomena and headache in occipital epilepsy: A review, a systematic study and differentiation from migraine. Epileptic Disord..

[18] Lepore F.E. (1990). Spontaneous visual phenomena with visual loss: 104 patients with lesions of retinal and neural afferent pathways. Neurology.

[19] Sanchez-Ramos J.R., Ortoll R., Paulson G.W. (1996). Visual hallucinations associated with Parkinson disease. Arch. Neurol..

[20] Goodwin D.W., Alderson P., Rosenthal R. (1971). Clinical significance of hallucinations in psychiatric disorders. A study of 116 hallucinatory patients. Arch. Gen. Psychiatry.

[21] Turner R.C., Lichstein P.R., Peden J.G., Busher J.T., Waivers L.E. (1989). Alcohol withdrawal syndromes: A review of pathophysiology, clinical presentation, and treatment. J. Gen. Intern. Med..

[22] Szucs A., Janszky J., Holló A., Migléczi G., Halász P. (2003). Misleading hallucinations in unrecognized narcolepsy. Acta Psychiatr. Scand..

